# Techniques for Clutter Suppression in the Presence of Body Movements during the Detection of Respiratory Activity through UWB Radars

**DOI:** 10.3390/s140202595

**Published:** 2014-02-07

**Authors:** Antonio Lazaro, David Girbau, Ramon Villarino

**Affiliations:** Department of Electronic, Electric and Automatic Control Engineering, Universitat Rovira i Virgili (URV), Av. Països Catalans 26, Campus Sescelades, Tarragona 43007, Spain; E-Mails: david.girbau@urv.cat (D.G.); ramon.villarino@urv.cat (R.V.)

**Keywords:** breathing monitoring, sleep apnea, vital signs, ultra-wideband radar

## Abstract

This paper focuses on the feasibility of tracking the chest wall movement of a human subject during respiration from the waveforms recorded using an impulse-radio (IR) ultra-wideband radar. The paper describes the signal processing to estimate sleep apnea detection and breathing rate. Some techniques to solve several problems in these types of measurements, such as the clutter suppression, body movement and body orientation detection are described. Clutter suppression is achieved using a moving averaging filter to dynamically estimate it. The artifacts caused by body movements are removed using a threshold method before analyzing the breathing signal. The motion is detected using the time delay that maximizes the received signal after a clutter removing algorithm is applied. The periods in which the standard deviations of the time delay exceed a threshold are considered macro-movements and they are neglected. The sleep apnea intervals are detected when the breathing signal is below a threshold. The breathing rate is determined from the robust spectrum estimation based on Lomb periodogram algorithm. On the other hand the breathing signal amplitude depends on the body orientation respect to the antennas, and this could be a problem. In this case, in order to maximize the signal-to-noise ratio, multiple sensors are proposed to ensure that the backscattered signal can be detected by at least one sensor, regardless of the direction the human subject is facing. The feasibility of the system is compared with signals recorded by a microphone.

## 1. Introduction

Obstructive Sleep Apnea (OSA) is the most common form of sleep-related breathing disorder (SRBD). OSA is characterized by repetitive obstruction of the upper respiratory tract during sleep, resulting in oxygen desaturation and frequent awakening. Its clinical importance is increasingly recognized [1–4]. Sleep apnea affects sleep duration and quality, leading to chronic partial sleep deprivation, with the consequent widely acknowledged impaired neurocognitive function and daytime performance, increased risk of metabolic and cardiovascular diseases [1] (e.g., hypertension, coronary heart disease, life-threatening arrhythmias and strokes) and motor vehicle accidents [2], and diminished quality of life. Large prospective cohort community-based studies have demonstrated that sleep apnea increases risk of death [4]. Although OSA is a worldwide problem, affecting around 4% of men and 2% of women [4], the majority of affected individuals remain undiagnosed.

The standard diagnostic tool for OSA is Polysomnography (PSG), which measures a wide range of parameters, including brain waves (EEG), eye movements, skeletal muscle activation, ECG/heart rate, airflow, respiratory effort, and blood oxygen saturation using a wide range of sensors. However, PSG requires costly measurement devices and labor-intensive set-up work for the electrode hook-ups, which often also disturb sleep. The technique also involves lengthy analysis requiring highly specialized staff. There is therefore growing interest in alternative approaches to the diagnostic assessment of OSA.

Contact-type approaches include Respiratory Inductive Plethysmography (RIP) [4]. In this approach, the respiration rate is measured by measuring changes in the chest and/or abdominal volume by fastening belts around these areas. Thoracic-abdominal elastic belts measure changes in the body circumference during the respiratory cycle, detecting the change in the inductance of the belt due to the respiratory effort. This is an invasive technique for respiratory monitoring and this method requires accurate positioning and tightness of the belt. Other techniques are the electrocardiogram method [5], the nasal temperature probe [6], the contact-type microphone for audio analysis to monitor tidal volumes from human breathing activity [7], capacitive textile sensors [8] and pressure sensor arrays [9]. The main disadvantage of these technologies [4–7] is their physical contact with the patient, which in some cases may be quite uncomfortable or impractical. Moreover, contact-type approaches often fail to monitor continuously because the devices can be unconsciously moved by the patient during sleep. All these methods require special beds or mattresses which can affect the patient's sleep. The recording may therefore not be a true indicator of the patient's daily sleep patterns. Doppler radars have been used in wireless sensor applications for decades [10–13]. The first example of a radar-based vital sign monitoring system was developed in the mid-1980s [14]. Since then, there have been several works that report the utilization of Doppler radars for cardiopulmonary [10–12] and life detection to locate humans trapped in earthquake rubble [13]. On the other hand, vital sign monitoring using micropower impulse radar was proposed by McEwan [15]. Since then, the interest in ultra-wideband (UWB) radars for biomedical applications [16,17] is increasing. Since the legalization of UWB by the FCC in 2002, UWB technology has awaken great interest in wireless communication [18–20] and radar sensor applications [21–24]. This technology has unique features due to its extremely wide bandwidth. UWB wireless systems are generally based on the transmission and reception of sub-nanosecond pulses without carriers, or on the transmission of modulated short pulses with carriers. These wireless systems are simple, cheap, low-power and permit high data rates [18,19]. There are also many advantages of using this technology for biomedical applications [16,17,21,22]. Since UWB technology radiates and consumes low power, it works well with other instruments, and it is robust to narrowband and multipath interferences due to its large bandwidth [18]. UWB monitoring of breathing and heart rate has been studied in [15,25–36] as an alternative to Doppler-based systems. From the pioneering work presented by McEwan [15] some other works ([15,25–27,31,32]) have shown the feasibility of estimating the breathing rate using a wireless, contactless and non-invasive respiratory monitoring system based on low-cost commercial UWB radars that can be used in PSG studies and home respiratory applications. An analytical framework to analyze respiration rate estimation from a received UWB waveform has been presented in [27]. Other works have been focused in the detection of breathing and heart rate simultaneously using UWB radars [25,31,32]. These works show that the estimation of heart beat is a challenge because it could be masked by two undesirable phenomena: the noise and the intermodulation between the breathing and the own heart signals [35]. In order to reduce these interferences due to the harmonics of the breathing signal on the heart signal, a harmonic filter has been proposed in [35]. The interest of contactless monitoring systems using both radar systems has increased in the last years. Using UWB or Doppler radars, the breathing signal can be detected with reasonable accuracy even behind walls [28,29,35], which is especially important in rescue applications. Furthermore, wearable UWB sensors have been proposed for vital sign monitoring [30] and body area network applications [37]. The main drawback of UWB radars for vital sign monitoring is the lack of low-cost integrated circuits (IC), whereas Doppler radars can be implemented using standard microwave components. Due to the lack of UWB ICs the first demonstrations often used hybrid microwave solutions [15,31,32] or high-cost laboratory equipment such as wideband oscilloscopes and pulse generators [27]. Nowadays the situation has changed. Commercial development kits [38,39] have appeared in the last years. Several groups have recently developed CMOS integrated circuits that implement UWB radar solutions for vital sign monitoring [30,33,34,40].

However, there are some open challenges to which this manuscript intends to contribute. Long-duration signals must be analyzed in apnea studies. Some random motions can therefore affect the breathing rate estimation. These intervals must be detected in order to mitigate these sources of errors. Furthermore the amplitude of chest displacement changes with time. As a result, when detecting the event when breathing ceases, a dynamic method to estimate the decision threshold is required. A previously proposed UWB system for monitoring breathing [35] uses two antennas pointed towards the chest-one for transmission and the other for reception. The performance of these systems depends on their orientation [41–43], but unfortunately they are also sensitive to the movement of the subject. Since one fixed antenna is used, if the human subject is not facing the antenna at a sufficiently small angle, the backscattered signal comes mostly from the side of the body and not from the chest area, resulting in poor estimation accuracy [43]. During sleep, the patient may turn and sleep on his side or backwards. The major challenge for a practical application of night breathing monitoring system is the noise signal produced by the body motion. Multi-static radars have previously been proposed for target tracking problems and localization (see for instance [44,45]). The main contributions of the paper are described below. First, a moving averaging filter to dynamically remove quasi-static clutter to enhance the breathing signal detection is proposed. Second, inspired in multi-static radar techniques, a setup comprising multiple UWB transceivers to solve the problem of body orientation is proposed. Multi-static Doppler radars have been proposed to cancel random body movement [46] using two radars, one at the front and the other one at the back. Multiple antenna input and multiple antenna output (MIMO) systems have been proposed in [47–49]. Therefore the use of MIMO processing techniques allows one to estimate cardiopulmonar frequency rates. Similar configuration but combining Self-Injection-Locked (SIL) oscillators and MIMO techniques [50] have been proposed for Doppler radar monitoring. Differential front-end Doppler radar operating at two different frequencies [51] is used in order to reduce the influence of body motion. However, these configurations may be difficult to install for overnight breathing monitoring if the person is lying in the bed. In this case, only Doppler based radar systems can be applied. Random body motion compensation in UWB monitoring systems has been proposed by the authors in [52] and it has also been investigated by others in [53]. The main idea is to estimate the displacement, aligning the received pulse waveform with a previously recorded waveform used as reference. The alignment is performed computing the cross-correlation between the signals. By determining the peak of the cross-correlation it is possible to estimate the delay between the signals and thus the distance. Our experience demonstrates that the method works well if the orientation of the body does not change, but if the patient eventually turns over in the bed, the waveform changes and the correlation falls. The third contribution is a simple algorithm for random body motion detection. After detected, these time intervals are not taken into account for breathing rate estimation. To this end, a robust spectral estimation algorithm compatible with variable sampling periods such as the Lomb periodogram is used. Once the random body motions are suppressed, the apnoea intervals can be determined using an adaptive threshold.

In case of UWB radar respiration detection, the movement can be detected without the necessity of multiple radars, although the use of diversity techniques can help to improve the detection. One transmit and two receive antennas are placed at different locations to ensure the detection of the backscattered signal by at least one receiver antenna. This simple configuration only needs one UWB radar if its UWB receiver input is connected to the receiver antennas using a microwave switch. A simple selection combining diversity technique has been implemented. In contrast with other MIMO systems proposed in Doppler radar systems, here the configuration of the antennas is compatible with bed-based night-time environment monitoring applications because the antennas are located in front of the patient. An algorithm to select the channel receiver with better signal-to-noise ratio in the frequency band of the breathing spectrum is proposed. Furthermore, unlike other numerical approaches [47–49], this technique is simpler than others previously proposed.

The paper is organized as follows: Section 2 presents and discusses the proposed measurement setup used to obtain the experimental results. Section 3 describes the mathematical formulation of the problem and signal processing techniques used for clutter suppression, motion detection and breathing rate estimation. Section 4 presents some experimental results for different cases. Finally, Section 5 offers the conclusions.

## 2. UWB RADAR Setup

**Figure 1.**
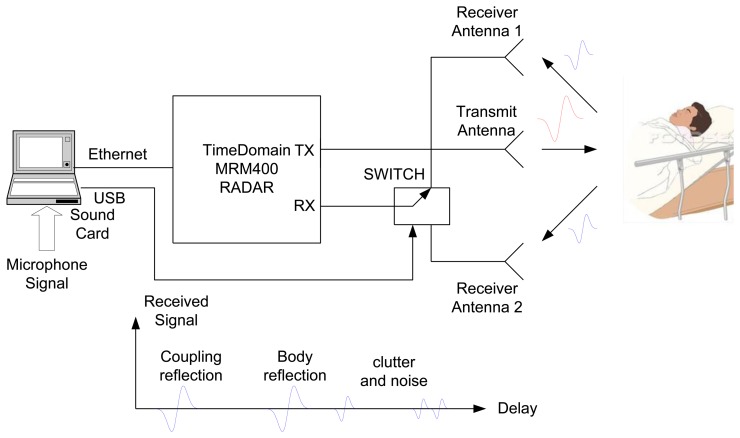

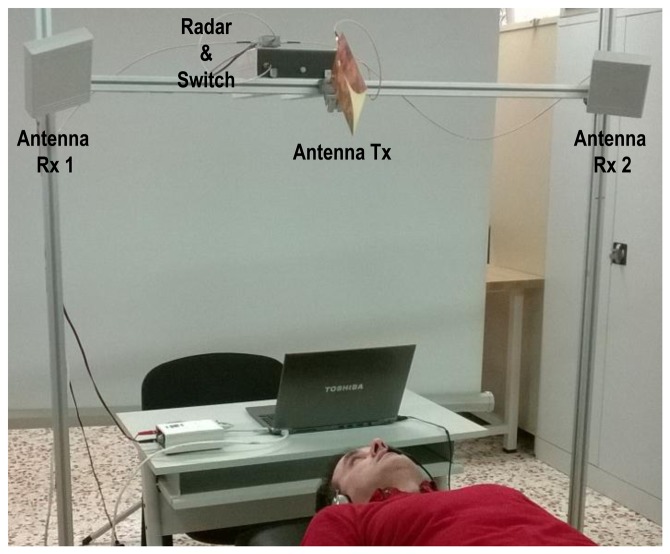
UWB radar test setup and photography.

**Figure 2.**
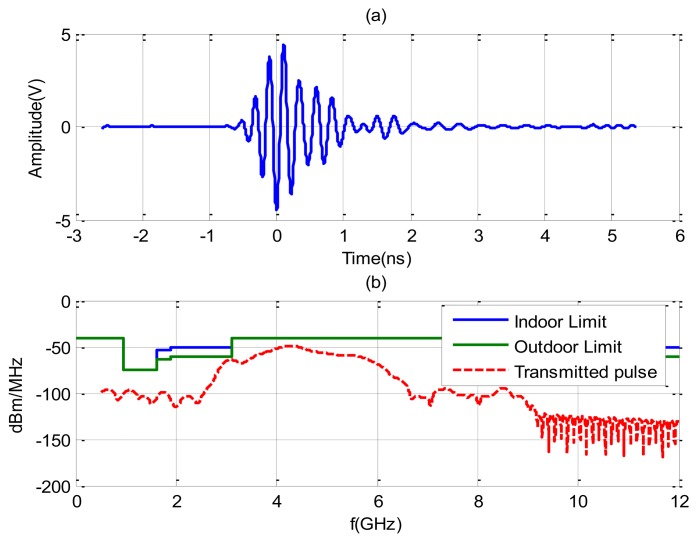
(**a**) Measured transmitted pulse in time-domain; and (**b**) the power spectrum density (PSD) of UWB transmitted pulse and FCC indoor and outdoor masks.

The experiments are performed using the NRM400 UWB radar from TimeDomain [39], which is briefly described here. A block diagram and photography of the measurement system to reproduce a typical scenario (at home or in apnea diagnosis) is shown in Figure 1. The distance between the body and the antennas is about 1 m and the distance between transmitting antenna and receiving antennas is 65 cm. The center frequency of the radar is around 4.3 GHz and its bandwidth is 1.3 GHz. The pulse repetition frequency (PRF) is 10 MHz and a coherent integration scheme of pulses is used (4,096 pulses). Figure 2 shows the typical waveform generated by the radar (Figure 2a). Figure 2b shows the output spectrum. It can be shown that the radar complies with the FCC frequency mask if the transmit power is adjusted to −14.5 dBm by choosing the correct gain of the internal amplifier of the radar. The delay resolution is 69.7 ps. The time interval for the data transfer between waveforms is limited to 14 Hz (7 Hz per channel). For simplicity, a Vivaldi UWB antenna with frequency range from 1–10 GHz is used in transmission and two commercial UWB antennas from Geozondas model AU-3.1G10.6G-1 (with a frequency range of 3.1–10.6 GHz) are pointed directly towards the subject for reception. The AS186-302 switch from Skyworks with a typical insertion loss of 1 dB at 4 GHz is used to switch between the two receiver antennas. The switch is controlled by a USB serial port using a PIC microcontroller. Therefore, the utilization of the switch allows the use of only one radar. The transmit antenna is located pointing to the center of the bed, and the receiver antennas are located 45 degrees at each side of the bed. The horizontal half-power beam width of the transmit antennas is about 100° and the horizontal half-power beam width of the receiver antennas are about 85° (directivity of 9 dBi). This configuration of antennas allows illuminating the whole bed for typical distances between 1 m to 3 m.

In order to improve the signal-to-noise ratio, 4,096 waveforms are acquired, sampled and averaged. The averaged waveform is stored and time-gated to avoid the coupling of signals between the transmitting and the receiving antennas. The waveforms are sampled at 14.3 GHz and the recorded duration is 45 ns (although this varies depending on the distance between the sampler and the subject). Reflections due to objects which are a long way from the antennas are automatically removed by the time window. The coupling between transmitting and receiving antennas and other static objects is removed using the clutter removing algorithm described in next section. The time axis associated with the range along each received waveform (*τ*) is denoted as “fast-time”, and is in the order of nanoseconds. Since the radar is composed by an only receiver, the signals collected by the two receiving antennas are interleaved. The process consists on sampling the signal received by the antenna 1, then the receiver is switched to the other antenna and the signal received by the antenna 2 is sampled. Then, the switch changes to the first antenna and the procedure is repeated. The interval between successive received waveforms is *T_s_* = 1/7 s. The time axis along the measurement interval (*t*) is denoted as “slow-time”, and is in the order of seconds. This means that the sampling frequency in slow time, *F_s_* = 1/*T_s_* = 7 Hz, is greater than the Nyquist sampling rate for the breathing signal (bandwidth <0.7 Hz). In order to compare the periods of apnea with an independent system, the signal from a microphone placed near the nose is sampled simultaneously with a computer sound card to monitor breathing. The microphone can detect breathing even if the person moves but it is an invasive system that can be uncomfortable during sleep. Therefore, it will be only used for comparison purposes.

## 3. Theory

### 3.1. Breathing Signal Detection

Disordered breathing is characterized by deviations in either amplitude or breathing rate from normal breathing, e.g., shallow or non-existent breathing (apnea) or rapid breathing (hyperpnea) [3]. In order to evaluate these disorders, the breathing frequency and the apnea periods must be detected. The spectrum of the detected signal is obtained in [35]. Both harmonics and cross-products (intermodulation) can be analyzed using the model described in [35]. Some aspects will be revised here for completeness.

When the transmitted pulse hits the patient, part of it is reflected due to the high reflectivity of the body. The time-of-flight or time-of-arrival (ToA) of this pulse, denoted by *τ_0_*, depends on the nominal radar distance *d_0_*. Due to breathing and heart motion, the chest cavity expands and contracts periodically, meaning that the distance travelled, *d*(*t*), varies periodically around the nominal distance *d_0_*. The signal received can be represented as the sum of the responses of the channel and the variation due to breathing:

(1) $$r(t,\tau )=\sum_{i}A_{i}p(\tau -\tau_{i})+Ap(\tau -\tau_{d}(t))$$

where *p*(*τ*) is the normalized received pulse, *A_i_* is the amplitude of each multipath component, *τ_i_* its delay, and *A* is the amplitude of the reflected pulse on the body that depends on the body radar cross section (RCS) and the body orientation.

The time delay *τ_d_* (associated with vital signs) is modeled as the sum of the ToA (*τ_0_*) plus two delays associated with a periodical chest movement due to breathing (with a frequency *f_b_*) and heartbeats (with frequency *f_h_*):

(2) $$\tau_{d}(t)=2d(t)/c$$

(3) $$d(t)=d_{0}+m(t)=d_{0}+m_{b}\sin(2\pi f_{b}t)+m_{h}\sin(2\pi f_{h}t)\approx d_{0}+m_{b}\sin(2\pi f_{b}t)$$

where *c* is the speed of light, and *m_b_* and *m_h_* are the breathing and heart amplitude, respectively. The peak-to-peak chest motion due to respiration in adults ranges from 0.4 to 1.2 cm [41]. Thus, in this study, only the breathing signal is considered because the heart amplitude is considerably lower than the breathing amplitude [42]. In addition, the heartbeat signal can be filtered because the frequency range varies between 1–2 Hz, whereas the frequency range of breathing signal is between 0.1–0.7 Hz.

The received waveforms *r* are measured periodically in slow time *t* = *nT_s_* (*n* = 1,2,…,*N*). *N* discrete-time sequences are stored after the received signal is sampled. These values are stored in a matrix *R*, the elements of which are:

(4) $$R[m,n]=r(\tau =mT_{f},t=nT_{s})$$

where *T_f_* is the sampling period in fast time.

In a static environment, the resulting clutter represents a DC component in the slow-time direction. In such an environment, the only movement is caused by the person's breathing and heart activity, and the background clutter does not depend on slow time *t*. The background clutter is removed by subtracting the average of all received waveforms from the original signal. The DC component is blocked by subtracting the average of all samples in fast time. More details about the clutter suppression will be done in the next section. The signal of interest *y* is recovered, after the clutter removal process:

(5) $$y(t,\tau )=Ap(\tau -\tau_{d}(t))$$

As *m_b_*≪*d_0_*, the breathing movement can be seen as a perturbation of the delay and it can be classified as a micro-movement (*m_b_* less than range resolution ΔR = c/(2B), where B is the pulse bandwidth and *c* the light velocity). The signal *y* can then be expanded in a Taylor series around *τ* = *τ_0_* = 2*d_0_*/*c*:

(6) $$y(\tau ,t)=Ap(\tau -\tau_{0})+Ap^{\prime }(\tau -\tau_{0})(-m_{b}\sin2\pi f_{b}t)+A\frac{1}{2}p^{\prime \prime }(\tau -\tau_{0}){\left(-m_{b}\sin2\pi f_{b}t\right)}^{2}+\cdots$$

The first term in (6) represents the average reflection of the body. If the subject remains fixed, this term is constant. The second term is the main contribution of the breathing signal. The other terms result on harmonics of breathing signal. In this work, the breathing signal is obtained by finding the maximum of *y*(*τ*,*t*) as a function of the fast time index:

(7) $$x(t)=\underset{\tau }{\max}y(\tau ,t)\approx y(\tau_{0},t)=Ap(0)-Ap^{\prime }(0)m_{b}\sin(2\pi f_{b}t)$$

It is assumed that the maximum occurs for *τ* = *τ_0_* in spite of the pulse waveform dependence. The DC term *Ap*(0) can be removed by subtracting the average signal over slow time index. Note that (7) can be used to follow sleeping that is stationary or semistationary (sleeping with little movement) because it is not necessary to know the average distance *d_0_*.

**Figure 3.**
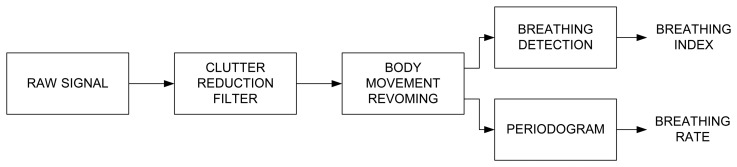
Signal processing block diagram.

The signal processing consists of the three main steps, described in Figure 3. These steps are: 1/Clutter reduction (see Section 3.2), 2/Body movement removing (see Section 3.3) and 3/Breathing signal detection and Spectrum estimation (see Section 3.4).

### 3.2. Clutter Reduction

In this application, the position of the human body is assumed to remain fixed in bed, except when the body sometimes changes its position. Clutter is therefore assumed to be basically stationary and can be removed using the background subtraction method. For simplicity, we only consider a given range cell. Let *r*(*n*) denote a column of the measured matrix R (4). The background subtraction method consists of subtracting an estimation of the background of *r*(*n*) [54] from the signal:

(8) y(n)=r(n)−b(n−1)

where *b*(*n-1*) is the background estimated from the average of *M* samples. The basic estimator is the moving average method, where the background is estimated as the average of previous *M* samples:

(9) $$b(n-1)=\frac{1}{M}\sum_{j=n-M}^{n-1}r(j)=(1-1/M)b(n-2)+r(n-1)/M$$

Note that the estimated background can also be expressed in a recursive form. After some manipulations and z transformation, the transfer function can be obtained:

(10) $$H(z)=Y(z)/R(z)=\frac{1-z^{-1}}{1-\lambda z^{-1}}$$

where *λ* = 1 − 1/*M*. The most simple case is to consider *M* = 1, where the background is assumed to be equal to the previous sample.

An improvement in the basic moving averaging consists in the use of a weighting factor. When the weighting factors follow an exponential law, this is known as exponential moving averaging [36]. This method uses all historical pulses to estimate the background data:

(11) b(n−1)=b(n−2)+(1−α)y(n−1)

where 0 ≤ α ≤ 1, controls the averaging. This method emphasizes recent events and gradually attenuates past data. After the same operations, it can be shown that the transfer function for the exponential averaging can be obtained from (10), but using *λ* = *α*. The exponential averaging can therefore be seen as a particular case of moving averaging, but with a sliding window with a fractional length of 1/(1-*α*).

**Figure 4.**
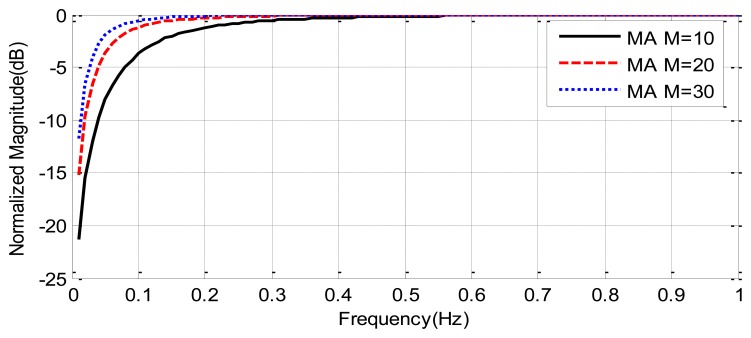
Frequency response of the moving averaging filter with different window lengths, *M*.

Figure 4 compares the frequency response of moving average filters as a function of the tap-length. It can be shown that the attenuation of clutter decreases with the tap-length, but the cut-off frequency also decreases with the tap-length. A tap-length *M* = 30 (about 4 s of window duration) is a good compromise between clutter attenuation and a cut-off frequency below the frequencies of the breathing signal (0.1–0.7 Hz).

### 3.3. Body Movement Removing

The movement of the chest due to breathing can be considered a type of micro-motion. The human target therefore remains in the same range cell. Any body movement or twitch during recording may cause a great distortion in the breathing signal. A random body movement, e.g., a change in the position during sleep, causes a macro-motion. If this occurs, the human target falls in several range cells. During such intervals, the respiration rate may be barely detectable. Before identifying the respiration signal, the artifacts caused by body movements are removed using a threshold method. The motion is detected using the time delay that maximizes the received signal after clutter removing algorithm:

(12) $$\tau_{m}(t)=\underset{\tau }{\arg\max}y(\tau ,t)$$

The human range is estimated as: *d*(*t*) = *cτ_m_*(*t*)/2. The sliding standard deviation using a moving window of length *L* = 30 (about 4 s) is then computed:

(13) $$\sigma_{d}(n)=\sqrt{\frac{1}{L}\sum_{i=n-L+1}^{n}d^{2}(i)-{\left(\frac{1}{L}\sum_{i=n-L+1}^{n}d(i)\right)}^{2}}$$

If the moving deviation (13) is higher than a threshold distance, it is considered a movement by the patient, and these samples are discarded and not used for the estimation of the breathing rate. Since the chest displacement is typically smaller than 2.5 cm, the threshold distance is chosen equal to 2.5 cm.

### 3.4. Breathing Signal Detection and Spectrum Estimation

Unfortunately, *x*(*t*) can be contaminated by noise and residual clutter. A robust spectrum estimation technique must therefore be used in order to estimate the breathing rate from *x*(*t*). In this study, the Lomb periodogram method is used [55,56]. The Lomb spectrum has been used in the study of physiological signals such as heart rate because it can clearly show the very low frequency components of the instantaneous heart rate [56]. It has been found to produce more robust power spectrum density (PSD) estimates in heart rate variability analysis compared to autoregressive (AR) and FFT methods in the presence of noise [57]. For time-series of N data points *x_j_* = *x*(*t_j_*) collected at times *t_j_* where *j* = 1,2…*N*, with a mean of x̄, the Lomb periodogram was computed from:

(14) $$P_{x}(\omega )=\frac{1}{2}\left(\frac{{\left[\underset{j}{\text{∑}}\left(x_{j}-\bar{x})\cos\omega (t_{j}-\tau_{x}\right)\right]}^{2}}{\underset{j}{\text{∑}}\cos^{2}\omega (t_{j}-\tau_{x})}+\frac{{\left[\underset{j}{\text{∑}}\left(x_{j}-\bar{x})\sin\omega (t_{j}-\tau_{x}\right)\right]}^{2}}{\underset{j}{\text{∑}}\sin^{2}\omega (t_{j}-\tau_{x})}\right)$$

where the delay *τ_x_* is obtained using:

(15) $$\tau_{x}=\frac{1}{2\omega }\tan^{-1}\left(\frac{\underset{j}{\text{∑}}\sin2\omega t_{j}}{\underset{j}{\text{∑}}\cos2\omega t_{j}}\right)$$

A fast implementation of the Lomb periodogram described in [57] is used in this study. As the periodogram is computed using a running window, the computational load and memory required are small. One advantage of this Lomb periodogram is that it can be used on evenly sampled data with periods of missing sections. The algorithm then selects the frequency with the highest amplitude associated with the respiratory component.

In order to detect apnoea, the breathing index (BI) is defined as 1 if the power of the breathing signal is higher than a decision threshold *σ_th_*, and zero otherwise, in this last case assuming that the patient is not breathing and that the signal power hence consists of residual clutter and noise.

(16) $$BI=\{\begin{array}{cc} 1, & \sigma_{x}>\sigma_{th}\\ 0, & \sigma_{x}\leq \sigma_{th} \end{array}$$

where *σ_x_* is the smooth average deviation of the signal *x*(*t*) and it is computed using a window of length *L* in a similar way to (13):

(17) $$\sigma_{x}(n)=\sqrt{\frac{1}{L}\sum_{i=n-L+1}^{n}x^{2}(i)-{\left(\frac{1}{L}\sum_{i=n-L+1}^{n}x(i)\right)}^{2}}$$

**Figure 5.**
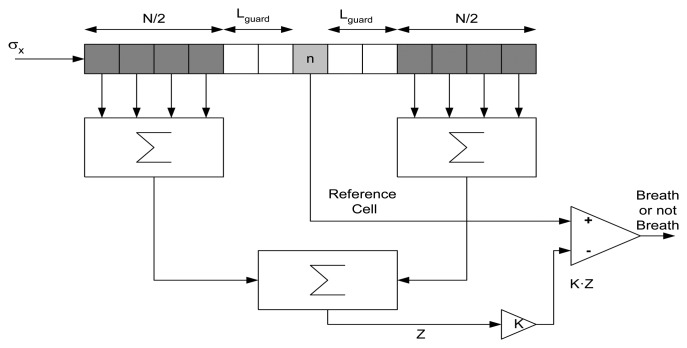
CFAR processor for breathing detection.

For long time series, it is expected that the amplitude of the breathing signal during respiration will change. An adaptive threshold limit will therefore be estimated. In existing CFAR systems, the target decision is commonly performed using the sliding window technique [58]. The data available in the reference window is used for the calculation of the decision threshold by an algorithm. This procedure is illustrated in Figure 5. The first step is to measure the mean clutter power level *Z*. The decision threshold for each time sample *n* is then obtained by multiplying this estimation *Z* by a scaling factor *K*, depending first of all on the estimation method applied and second on the false alarm rate required. The resulting product *K·Z* is directly used as the decision threshold value, *σ_th_*^2^. The clutter power is calculated using:

(18) $$Z(n)=\frac{1}{N/2}\sum_{i=n-N/2-L_{\text{guard}}}^{i=n-L_{\text{guard}}}\sigma_{x}^{2}(i)+\frac{1}{N/2}\sum_{i=n+L_{\text{guard}}}^{i=n+L_{\text{guard}}+N/2}\sigma_{x}^{2}(i)$$

where in (18), *N* is the size of the window used in the averaging, and *L_guard_* is the number of guard cells around the reference sample n left to prevent edge effects. In this work, *N* is set to 30, *K* = 0.5, and *L_guard_* = 1.

In order to avoid problems associated with the orientation of the antennas to the body, multiple channels with different orientations can be recorded. The channel with best orientation will achieve a higher breathing signal than other channels. The channel with the highest signal to clutter noise ratio is then chosen. The breathing rate is estimated for each channel as the frequency *f_max_* of the peak in the periodogram *P_x_(f)*. The signal to noise clutter ratio (SCR) is estimated from the periodogram as the ratio between the power around the maximum of the periodogram and the power outside:

(19) $$\text{SCR}=\frac{\int_{f_{\max}-B/2}^{f_{\max}+B/2}P_{x}(f)df}{\int_{0}^{\infty }P_{x}(f)df-\int_{f_{\max}-B/2}^{f_{\max}+B/2}P_{x}(f)df}$$

where *B* = 0.05 Hz is the resolution in the estimation of the periodogram.

## 4. Experimental Results

**Figure 6.**
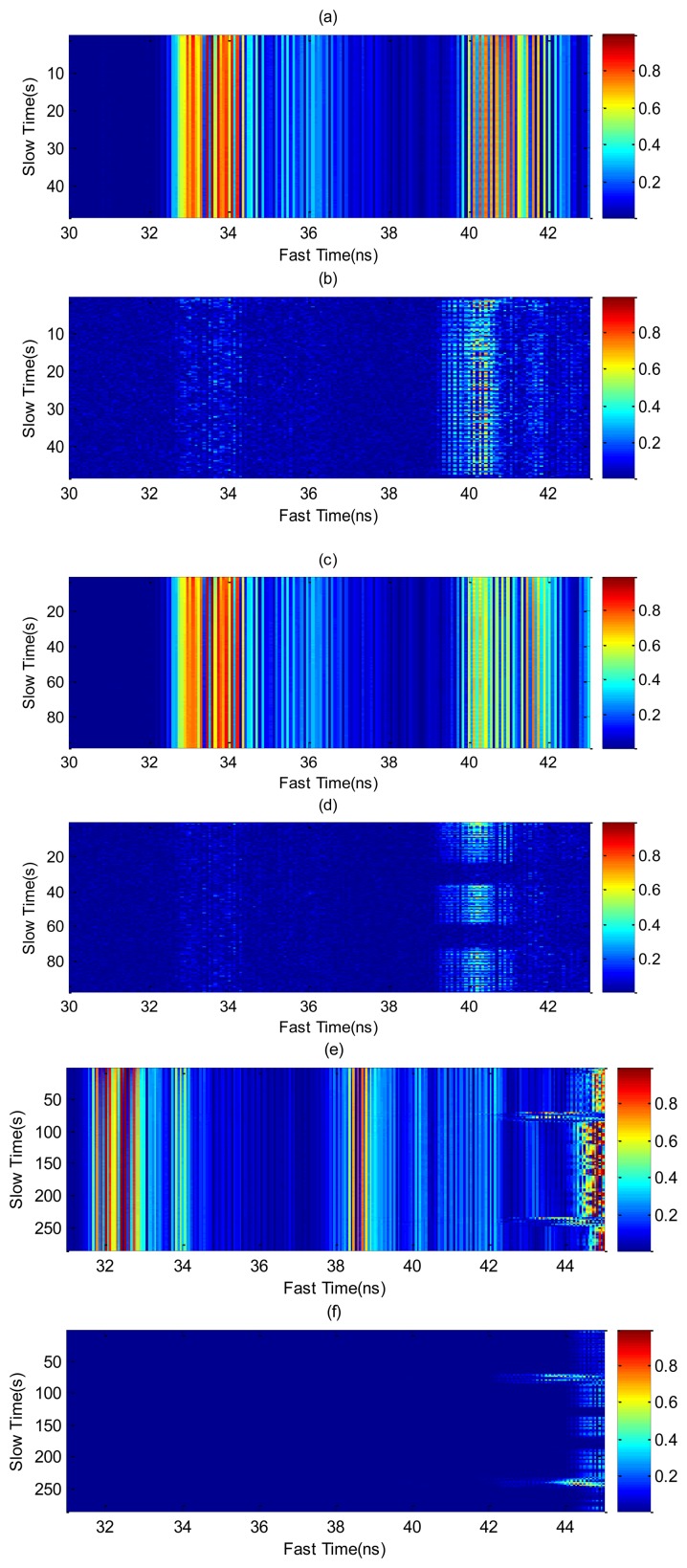
Raw Data and Data after clutter removing algorithm. (**a**) and (**b**) are from the case of a subject with continuous breathing, (**c**) and (**d**) are from the case of a subject with simulated periods of apnoea, and (**e**) and (**f**) are from the case of a subject with simulated periods of apnoea and random body movements.

In this section, some results for different cases are experimentally obtained using the setup shown in Figure 1. In order to investigate typical situations over a long-term monitoring the following measurements are performed on a young (22 year old) male volunteer who simulates several positions and cases. Figure 6 shows the raw signal, before and after clutter removal, measured by one of the receiver channels for different typical cases. The first case involves a static person who breathes continuously (Figure 6a,b). The high coupling between the transmitter and the receiver antenna is practically removed around 33–34 ns and the periodic chest displacements are visible in the slow time domain. Figure 6c,d shows the same result, but with the subject in repose with normal breathing and a simulated apnea. The apnea periods are detected after clutter removal (Figure 6d). Figure 6e,f shows the case where the subject performs a random movement. Unlike the previous cases, where the displacement of the chest corresponds to micro-movements, the movement of the subject causes macro-movements. The moving average method with *M* = 30 has been applied to remove the clutter contributions in all cases.

**Figure 7.**
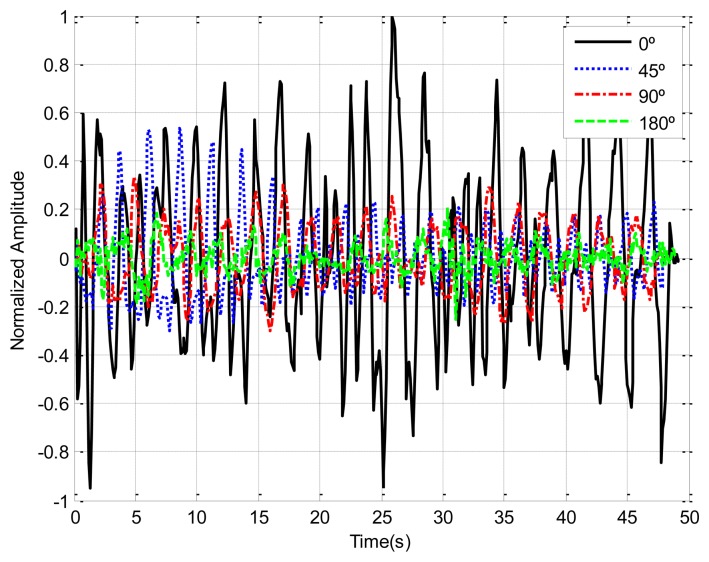
Dependence of breathing signal as function of orientation at a distance of 1 m.

Human body responses due to breathing motion are expected to depend on body orientation. The chest displacement is higher when the antenna is pointed at the patient's face than in other cases (back or side orientation). In order to study the dependence of the orientation between antennas and the chest patient, Figure 7 shows the recorded breathing signals for different orientations. In this measurement the transmitter antenna (Vivaldi antenna) and receiver antenna (Geozondas model AU-3.1G10.6G-1) are spaced 65 cm and oriented to the body at 1 m of distance. The amplitudes are lower and noisier for side and back orientation than for facing orientation. A multichannel system can therefore be used to mitigate this problem.

**Figure 8.**
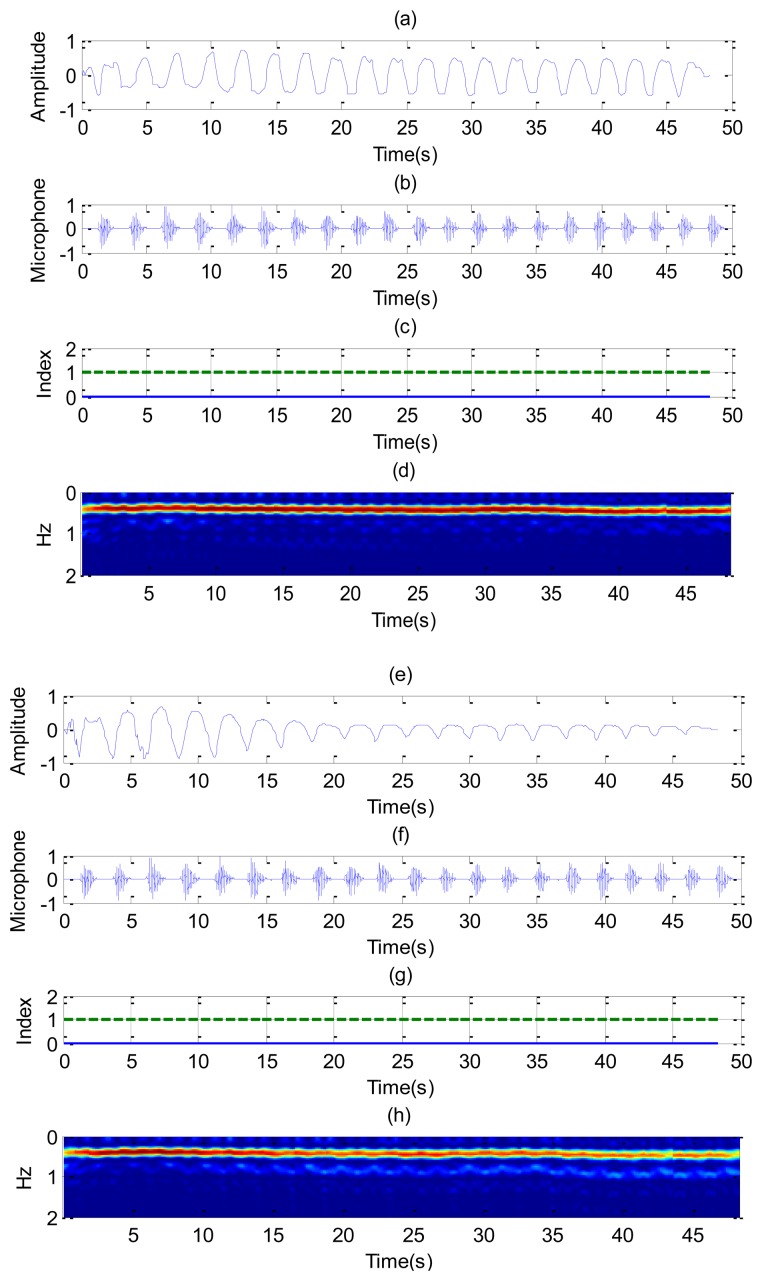
Results for a continuously breathing subject (case Figure 6a,b), channel 1 (left), and channel 2 (right). For the channel 1: (**a**) Breathing signal, (**b**) recorded signal from a microphone, (**c**) movement index (solid line) and breathing index (dashed line), (**d**) periodogram obtained from channel 1. For the channel 2: (**e**) Breathing signal, (**f**) recorded signal from a microphone, (**g**) movement index (solid line) and breathing index (dashed line), (**h**) periodogram obtained from channel 2.

**Figure 9.**
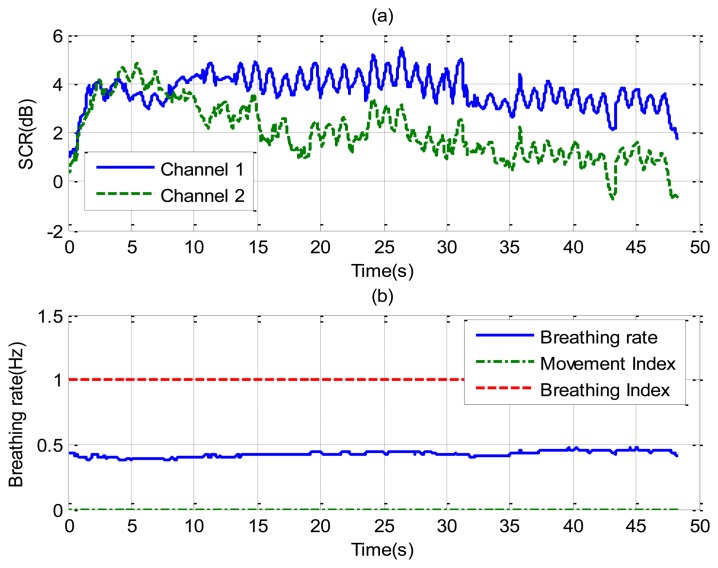
(**a**) Signal to clutter ratio as a function of time for each channel; (**b**) Estimated breathing rate as function of the time (-), movement index (--) and breathing index (-.), for the case of Figure 6a,b.

Figure 8 shows the recorded signal *x*(*t*) for two receiver channels for the case of a subject in repose with normal breathing (the case of Figure 6a,b). In this case, channel 1 is oriented to the side and channel 2 to the face. The breathing signal is obtained from the maximum of the measured data as a function of the slow time after removing the background for each case. Figure 8a,e shows the normalized breathing signal referred to the maximum value for both channels. The signal recorded with a microphone (Figure 8b,f) is added below for comparison purposes. Figure 8c,g plots the movement index and breathing index computed from Equations (13) and (16), respectively. These indexes demonstrate that a static subject has been breathing continuously. Figure 8d,h shows the periodogram for the two channels. The maximum of the frequency can be shown to remain approximately constant for the two channels. After the calculation of the SCR using (18), the breathing signal, breathing index and movement index for the channel with higher SCR are selected (Figure 9).

**Figure 10.**
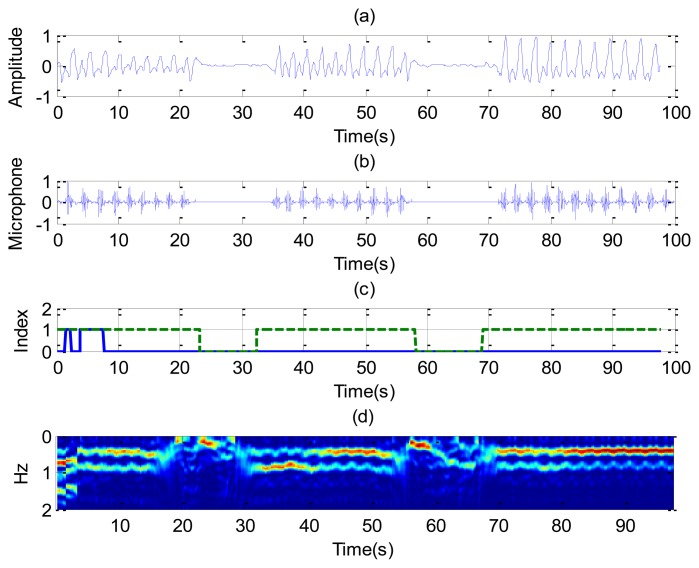

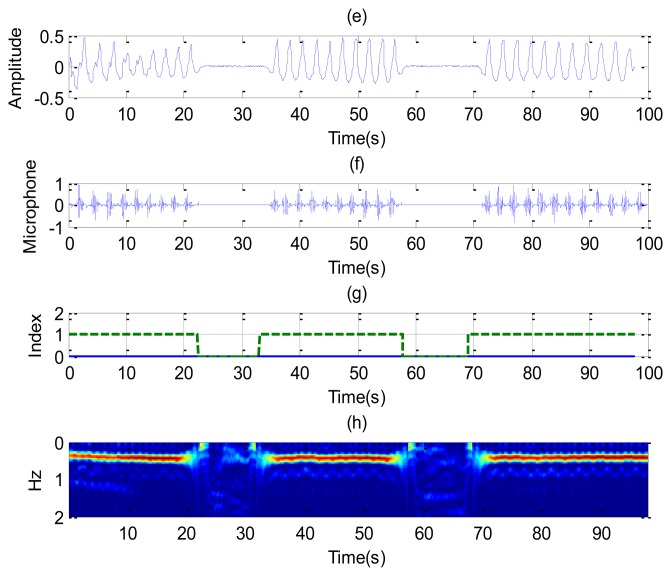
Results for a subject with simulated periods of apnoea (case Figure 6c,d), channel 1 (left), and channel 2 (right). For the channel 1: (**a**) Breathing signal, (**b**) recorded signal from a microphone, (**c**) movement index (solid line) and breathing index (dashed line), (**d**) periodogram obtained from channel 1. For the channel 2: (**e**) Breathing signal, (**f**) recorded signal from a microphone, (**g**) movement index (solid line) and breathing index (dashed line), (**h**) periodogram obtained from channel 2.

**Figure 11.**
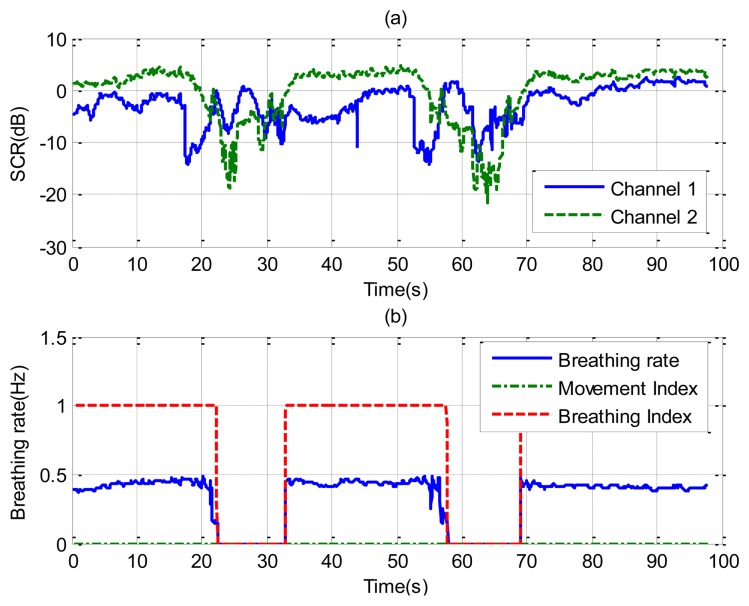
(**a**) Signal to clutter ratio as a function of time for each channel; (**b**) Estimated breathing rate as a function of the time (-), movement index (--) and breathing index (-.), for the case of Figure 6c,d.

Figure 10 shows the results for the case in which a static patient suddenly interrupts his normal breathing. Channel 1 is side-oriented and channel 2 is face-oriented. Comparison with the microphone signal shows that the apnea intervals are detected at both channels. The behavior of the SCR (Figure 11) is better for channel 2 than for channel 1 during intervals in which the patient breathes normally. The breathing rate is estimated from channel 2 in these intervals. From the results of Figure 10, it can be observed that sometimes the power level of the second harmonic is even higher than the level of the fundamental breathing frequency. This effect can be explained using Equation (6). The reflected pulse from the body is distorted by the body surface and the antennas. The amplitude of the second derivate term may therefore sometimes be higher than the first derivate term for the mean range *τ*_0_. When this occurs, the breathing signal presents a high level of the second harmonic. A similar conclusion can be drawn from the Fourier analysis presented in [35]. In practice, this is not a problem because the breathing frequency is obtained from the maximum in the breathing frequency range.

**Figure 12.**
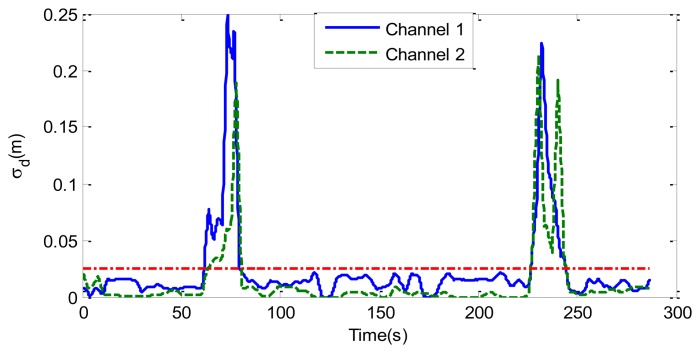
Standard deviation of the detected range as a function of the slow time for the two channels, and threshold limit (-.), for the case of Figure 6e,f.

**Figure 13.**
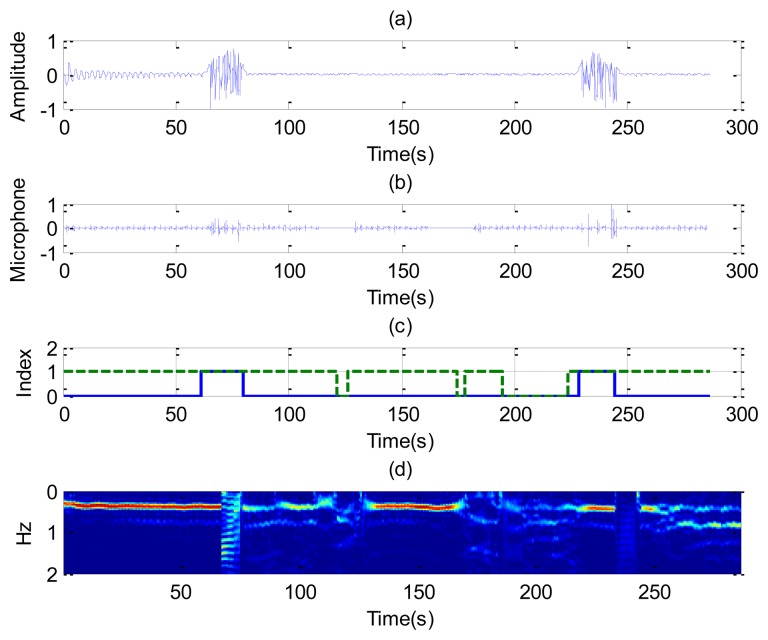

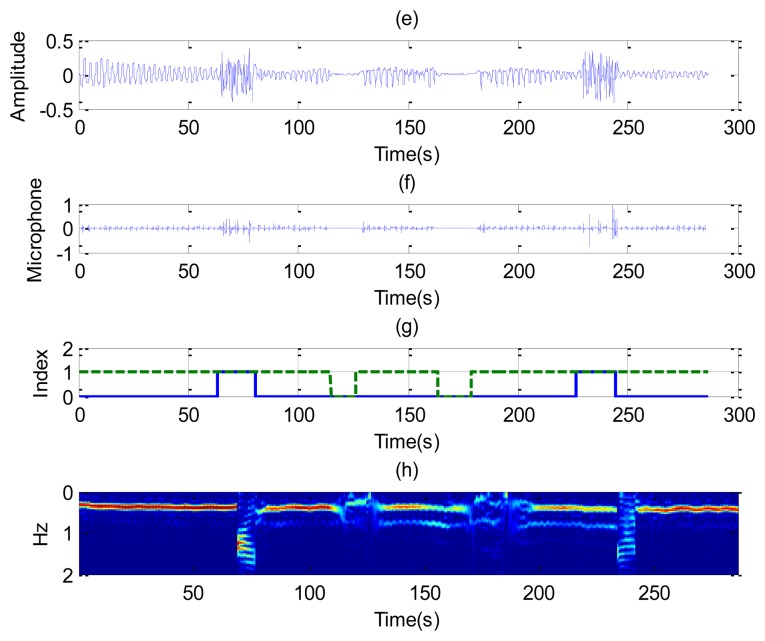
Results for a subject with simulated periods of apnoea and random body movements (case Figure 6e,f), channel 1 (left), and channel 2 (right). For the channel 1: (**a**) Breathing signal, (**b**) recorded signal from a microphone, (**c**), movement index (solid line) and breathing index (dashed line), (**d**) periodogram obtained from channel 1. For the channel 2: (**e**) Breathing signal, (**f**) recorded signal from a microphone, (**g**) movement index (solid line) and breathing index (dashed line), (**h**) periodogram obtained from channel 2.

**Figure 14.**
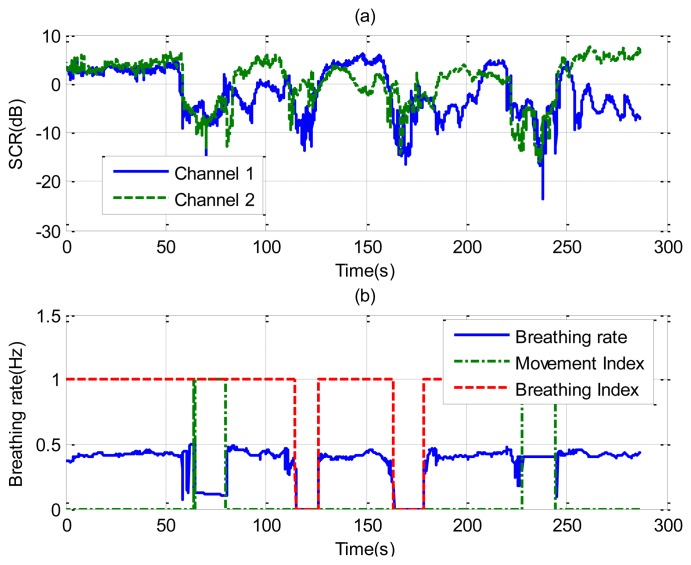
(**a**) Signal to clutter ratio as a function of time for each channel; (**b**) Estimated breathing rate as function of the time (-), movement index (--) and breathing index (-.), for the case of Figure 6e,f.

The last case considers random movement by the patient. Figure 12 shows the standard deviation of the range obtained from Equation (13) as a function of the slow time. The value is higher than the threshold limit of around 75 and 240 s, indicating a movement of the subject. A small change in the microphone signal is detected around these points. Figure 13 shows that the amplitude of the two channels is larger than normal breathing during motion intervals. It can be shown that the periodogram is nosier during the time intervals of random body movement than when the patient stops breathing. During these intervals, the spectrum presents different components and it is not possible to estimate the breathing rate. In these intervals the SCR falls (see Figure 14a). The first channel before the first motion is side-oriented and the second is face-oriented. The SCR is therefore higher for channel 2 than for channel 1. After the first movement, the subject orientation is the same. After the second movement the subject is oriented towards the middle of the two receiver antennas, and is therefore face-oriented for the two channels. The SCR is more similar to channel 2 than channel 1 when channel 1 is side-oriented.

## 5. Conclusions

This paper has presented a non-contact system based on an UWB radar to detect sleep-related breathing disorders such as sleep apneas. This work has proposed a method to detect movement by the subject. We show that it is difficult to estimate the breathing rate during motion periods. These intervals can be identified using a simple threshold technique thank to UWB's ability to estimate distances. These time intervals are of overall short duration, so they can be neglected for the breathing rate estimation since the Lomb periodogram technique is used. In contrast with other spectrum estimation techniques, this technique can be used with time samples that are not equally spaced, neglecting motion intervals. Application of the clutter suppression is fundamental in achieving the breathing signal with a good signal-to-noise ratio. Several approaches for clutter suppression have been reported in the literature. In this study, simple moving averaging has been used with good results for this application. It has been shown that the signal-to-noise-clutter is a function of the body orientation with respect to the antennas. The pulse waveform also depends on this orientation. In order to mitigate this problem, an antenna diversity system has therefore been proposed. Two receive channels, oriented at different angles, have been used. In a normal position on the bed, the two channels approximately detect the same signal when the body is facing the antennas. If the subject turns to one side, at least one of the channels is oriented to the face or the side, achieving good signal-to-noise-clutter- ratio from the chest motions. The breathing rate is obtained independently for each channel. The value of the channel with the best SCR is chosen at each instant. A CFAR technique is used to estimate the decision threshold. Moreover, the proposed system is based on low-cost UWB radar, and is suitable for long-term monitoring. The results obtained show that this technique may be particularly suitable for overnight sleep apnea monitoring, infant SIDS monitoring and home health care.
